# Factors Associated with Falls in Community-Dwelling Older Adults: A Subgroup Analysis from a Telemergency Service

**DOI:** 10.3390/geriatrics9030069

**Published:** 2024-05-29

**Authors:** Elena Casabona, Federica Riva-Rovedda, Angela Castello, Daniele Sciarrotta, Paola Di Giulio, Valerio Dimonte

**Affiliations:** 1 Department of Public Health and Pediatrics, University of Torino, 10100 Torino, Italy; elena.casabona@unito.it (E.C.); federica.rivarovedda@unito.it (F.R.-R.); angela.castello@edu.unito.it (A.C.); valerio.dimonte@unito.it (V.D.); 2 Assi.I.S.Te Company Scs, 10100 Torino, Italy; d.sciarrotta@assiste.it

**Keywords:** aged, personal emergency response system, fall detection device, recurrent falls, risk-profile, independent living

## Abstract

According to the number of falls, fallers can be single (only one fall) or recurrent (two or more falls), with different risk profiles for loss of independence and frailty. The presence of risk factors in community-dwelling single- and recurrent fallers using a wearable fall-detection device, such as the Personal Emergency Response System (PERS), as part of a telemergency service, is still unknown. This article evaluates how using a PERS, within a telemergency service, helps identify risk profiles and assessment of any differences between non-fallers and fallers in community-dwelling older adults. A sub-group analysis was performed, dividing users into non-fallers (n = 226) and fallers (≥1 fall; n = 89); single-fallers (n = 66) and recurrent fallers (n = 23). Median age was higher in fallers (87.7 years vs. 86), whereas recurrent fallers were less independent, had fewer comorbidities, and had more low-extremity disabilities. The use of the PERS for medical problems (Adjusted OR = 0.31), excluding falls, support calls (Adjusted OR = 0.26), and service demands (Adjusted OR = 0.30), was significantly associated with a fall risk reduction. The findings suggest that the integration within a telemergency service may impact on fall-risk factors.

## 1. Introduction

Thanks to the overall improvements in the standard of living, life expectancy is growing steadily [1]. Consequently, the older population is increasing worldwide, as are the conditions of ageing-related frailty. Among them, falls are one of the most concerning [2].

Falling is the most prevalent geriatric syndrome in older adults, defined as “an event which results in a person coming to rest inadvertently on the ground or floor or other lower level” [2]. Falls and related injuries are increasingly common and detrimental in the ageing population, making their prevention and management paramount [3]. Approximately 30% of individuals aged ≥ 65 experience a fall each year [4]. Even though they are common among clinical care settings, such as hospitals, nursing homes, and long-term care facilities [5], it has been reported that about one-third of home-dwelling adults fall at least once a year. Of those who fall, half are known as recurrent fallers, as they experience two or more falls per year [6]. Falls represent a global public health problem because of their consequences in terms of morbidity, mortality, and quality of life, as well as their impact on health care costs [7]. The most harmful consequences of injurious falls are hip fractures, brain damage, and death. Furthermore, in older people, falling can result in loss of confidence, emotional distress, fear of future falls, and loss of independence as the risk of institutionalisation increases compared to non-fallers [8]. The occurrence of a fall is a predictive factor of other falls in the future. The increasing number of falls, especially in short periods (i.e., recurrent fallers), decreases the functional response of older adults to stressors. Consequently, falls in older adults are associated with frailty, defined as a state of higher vulnerability, a decline in physiological reserves, and reduced tolerance to stressors [9]. In fact, factors associated with recurrent falls can be divided into seven domains: (1) balance and mobility; (2) environmental; (3) psychological; (4) medical; (5) medication; (6) sensory and neuromuscular; and (7) sociodemographic [10]. However, only four of these domains have been defined as makers of frailty (balance and mobility; medication; psychological; sensory and neuromuscular) [11].

The multifactorial aetiology of falls in older people has been widely recognised. The risk of falling is related to physiological changes that occur due to advanced age (e.g., sarcopenia), but also to the increase in the number of chronic conditions, polypharmacy, gait impairments, previous history of falls, high alcohol consumption, incontinence, cognitive and sensory impairments, and the fear of falling [5]. Moreover, older adults have an increased risk for social isolation, associated with a higher risk of many serious medical issues, delays in seeking medical treatment, reduced self-care and accidental injuries (i.e., falls) [9]. International guidelines advocate multifactorial assessment and interventions to address specific risk factors to prevent falls in older adults [5]. These interventions encompass strength and balance exercises, medication review, management of orthostatic hypotension and cardiovascular diseases, management of underlying acute and chronic diseases, treating visual and hearing impairments, addressing foot problems, choosing appropriate footwear, optimising nutrition, continence management, and environmental changes (e.g., removing potential home hazards), including the use of technology for environmental and gait monitoring [5]. Older adults who benefited from multifactorial interventions presented a reduction in the fall rate between 13% and 28% [12,13]. However, mixed evidence regarding a decrease in the number of people undergoing a fall was observed [12,13], as well as the effects of multifactorial interventions on fall-related outcomes, such as fractures, hospitalisations, and quality of life [14]. To be as effective as possible, multifactorial interventions should be tailored and personalised according to older adults’ characteristics and risk profiles [5].

Personal Emergency Response Systems (PERS) are among the most common technological devices available in fall management. They are accurate and cost-effective devices that can be worn around the neck or wrist and may promptly activate rescue systems by distinguishing a fall from normal daily activities [15,16]. Moreover, older adults use the PERS not only to communicate medical emergencies but also to request different kinds of support for health and non-health needs [17]. Considering its broader use, this device could become a valid instrument by improving the monitoring of older adults at home, enhancing independent living. Since recurrent fallers may be considered frailer than non- and single-fallers, the assessment of any differences in the needs reported through the PERS by older adults (excluding fall-related alerts) and the level of frailty could allow not only broadening of the service offer, but also provision of proactive care. To our knowledge, no previous studies reported the profile differences among non-, single- and recurrent fallers wearing a PERS nor the differences in the request for health and non-health needs.

Since fallers and non-fallers could present characteristics influencing their risk of falling, we hypothesised that fallers had more risk factors than non-fallers and those with a higher use of a PERS. This article aims to identify the risk profile and any differences between non-fallers and fallers, and between single- and recurrent fallers in a cohort of community-dwelling older adults who benefitted from a wearable telemergency system device offered by Ass.I.S.Te Cooperative (Ass.I.S.Te from now on) in the period between 1 December 2020 and 31 March 2023.

## 2. Materials and Methods

This is a sub-group analysis of a larger cross-sectional study of a telemergency service offered by a private company, in agreement with public authorities to promote independent living in older adults [17].

### 2.1. Study Setting

Ass.I.S.Te is a non-profit organisation that provides home care services for older adults and people with special needs. The telemergency service can be purchased privately or offered by public authorities (i.e., social or health services), which partially or fully cover its cost. The cost includes a monthly fee of 25 euros, plus the cost of activation and installation of the PERS (50 to 100 euros according to indoor-only monitoring, outdoor monitoring, or both) and a deposit (100 euros).

All the PERS belong to the third generation and are embedded with sensors (i.e., an accelerometer and a gyroscope) designed to automatically identify a fall or a high impact with a hard surface. The device or the user can generate an alert automatically or by pressing a button on the PERS. All the alerts are managed by a single centralised point of contact (TOC), with lay staff trained to better respond to the users’ needs. The user can communicate with the staff through the radio transmitter installed in all the PERS. The devices are also equipped with a call cut-off device in the event of a false alarm.

### 2.2. Participants

In the subgroup analysis, we included all public and private users who sent at least one real alert in the study period (1 December 2020 and 31 March 2023) and consented to processing of the information collected by the telemergency system for research purposes. Real alerts were defined as all active or automatically sent requests by the device for health and non-health problems. All alerts involving test alerts, technical issues, false (i.e., error from the users) or not-coded alerts were excluded from the analysis because they do not express a health or social need of the user.

The users included in this study are people residing in Italy who signed a contract for the remote assistance service with the Ass.I.S.Te agency (telemergency service) and received support during the study period.

Participants were divided into non–fallers and fallers; fallers were then divided into single–fallers and recurrent fallers.

### 2.3. Data Collection

Baseline information was extracted from the record collected by the TOC staff for each user. Among demographic information, sex (i.e., female), living condition (i.e., alone), and caregiver presence were considered as dichotomic variables; age was treated as a continuous variable, while marital status was categorised (i.e., single, married, divorced, and widowed). No scales were used to evaluate the health information (i.e., dependence level was deduced by analysing medical records from the TOC). The use of a walking aid, visual and hearing impairment, and lower limb disabilities were treated as dichotomic variables. Disabilities were considered as the presence of self-reported impairment, affecting partially or totally the use of a limb or a sense (i.e., hearing). Rather than a single diagnosis (i.e., heart failure), it is the number of comorbidities which increases the risk of falling; therefore, comorbidities were not categorised but considered as a continuous variable. Information on the number of drugs was collected, with a focus on increasing fall-risk medications (anticoagulants, antihypertensives, diuretics, analgesics, antidepressants, anxiolytics, hypnotics, antiparkinsonians, and neuroleptics) [15]. Lastly, the type of agreement was considered as a dichotomous variable (public and private). 

Twenty-eight months of alert data were downloaded from the software in use in the TOC. Since multiple real alerts could be sent for the same event (i.e., fall), alerts which identified the same events were aggregated independently by two researchers (E.C. and A.C.). Alerts reporting similar information (i.e., same date activation, closure date, and close alert timing) were aggregated and considered as one real event to prevent data misinterpretation and real event overestimation. Real events, other than falls, consist of medical problems (events involving cardiovascular or pulmonary symptoms, fever, malaise etc.), support calls (additional calls, compared to those performed two times weekly by the TOC staff, made by the user to alleviate a sense of loneliness), and service demands (user requests, such as for ambulance transportation, etc.). More information on the selection process was published in a recent article [17].

### 2.4. Statistical Analysis

Descriptive data are reported as absolute frequencies and percentages for categorical variables. The normality of the distribution of continuous variables was assessed with the Shapiro–Wilk normality test. Continuous variables with a non-normal distribution were presented as median and interquartile ranges (IQR). Differences between non-fallers and fallers, and between single- and recurrent fallers were assessed using the Chi-squared test for categorical variables and the non-parametric Wilcoxon rank-sum test for continuous variables.

A logistic regression model was performed to assess factors associated with falls (dependent variable) [18]. Sociodemographic characteristics, health information, and real events (medical problems, support calls, and service demands) were considered independent variables. Variables considered statistically significant (*p* < 0.05) in the univariate model were included in the multivariate regression model to adjust for the factors associated with falls.

Statistical analyses were performed using Jamovi Version 2.3.26.0 [19], and *p*-values < 0.05 were considered statistically significant.

## 3. Results

A total of 315 users were included in the study, 226 not experiencing a fall during the study period (non-faller group) and 89 at least one fall (fallers). Of the 89 fallers, 66 fell only once (single-fallers) and 23 fell at least twice (recurrent fallers).

### 3.1. Characteristics of Non-Fallers and Fallers

Differences between the two groups are shown in Table 1. Fallers and non-fallers were comparable for all the considered variables, except age. In fact, fallers were slightly older compared to non-fallers. Well-known risk factors for falls (i.e., female sex, loneliness, using of a walking aid, dependence level and lower-limb disabilities) were prevalent in the faller group, including a higher number of comorbidities, though not significant. Non-fallers significantly presented more medical problems (*p* = 0.02) and requested more support calls (*p* < 0.01) and service demands (*p* = 0.02) than fallers.

### 3.2. Factors Associated with Falls

In the univariate model, age was the only independent variable significantly associated with falls (OR = 1.03, 95% IC 1.002–1.056) (see Supplementary Table S1). Sex (female), the use of walking aids, and hearing and visual disabilities were not statistically significant in our model, nor the use of one or more fall-risk-increasing drugs. Older adults who requested support for medical problems and service demands and called the TOC for support calls presented a reduced risk of falling (Table 2). Adjusting for the confounders, users who needed support for medical problems and for services (service demands) were about 70% less at risk of falling, while considering only support calls, the risk decreased of 74%.

The total number of real events experienced was not associated with the risk of falling.

### 3.3. Characteristics of Single and Recurrent Fallers

Comparing single and recurrent-fallers, age was no longer significant (*p* = 0.78) (Table 3). On the contrary, recurrent fallers were significantly less independent, had fewer comorbidities, and more lower-limb disabilities. No differences emerged in the number of real events, except for medical problems (*p* = 0.01), more frequent in recurrent fallers.

A logistic regression model was not possible, due to the small number of users who experienced recurrent falls (n = 23).

## 4. Discussion

This study aimed to identify risk profile and any differences between non-fallers and fallers, and between single and recurrent-fallers in a cohort of community-dwelling older adults who benefitted from using a PERS within a telemergency system. Comparing sociodemographic and health characteristics, no major differences emerged between fallers and non-fallers: both populations were old (median age was ≥86 years). As underlined by Lage et al. [20], age itself is an important risk factor [21,22,23]. In both groups a sizeable proportion of patients had characteristics known in the literature for increasing the risk of falls, such as female sex, the use of walking aids, hearing and visual impairments as well as the use of fall-risk-increasing drugs [21,22,23]. These characteristics are highly prevalent in older populations and their interaction accounts for the risk of falls [21,22,23]. The abovementioned characteristics also contribute to a frailty phenotype [24] that acts as an additive risk factor for falls [25].

As already confirmed by the literature, falls and recurrent falls are sustained by a shared multifactorial aetiology, but not all falls are comparable events. Falls have been differentiated in four classes: accidental falls (falls occurred outside home), unanticipated physiologic falls (unknown intrinsic risk factor, i.e., seizures), anticipated physiologic falls (known intrinsic fall-risk factor, i.e., impaired gait), or intentional falls [26]. In our study, all the falls occurred at home. Considering the characteristics of our participants, these falls can be addressed to anticipated physiologic falls or the accidental category [26]. Considering the lack of differences in users’ intrinsic risk factors between faller and non-fallers, it can be deduced that all falls are predictable, but not entirely preventable and avoidable [27]. In general, other than prediction, prevention cannot completely avoid the event: even eliminating any environmental hazards in an older adult without any relevant fall-risk factor or impairment, experiencing an accidental fall is still possible.

Moreover, our findings suggest that the risk of falls is influenced not only by social and health factors, but also by older adults’ behaviours. Participants who used the PERS to request support for medical and non-medical problems, excluding falls, presented a reduced risk of falling. The request for more support, both for medical and non-medical needs, using a PERS could have helped non-fallers with avoiding falls and progressing through a frailty condition. As previously reported, PERS are often used equally for both signalling falls or medical problems (i.e., feeling unwell, hypotension etc.). In fact, Andrew et al. [28] underlined that nearly 40% of falls that occurred during the observation could have been a result of an acute medical problem. From our analysis, users who performed more calls for medical problems, though being comparable for the main characteristics, presented a fall risk reduction of 69% (Adjusted OR = 0.31; 95% CI 0.16–0.61). Most common medical emergencies, directly communicated by the users, in our study were cardiovascular symptoms (i.e., hypotension, tachycardia), respiratory symptoms (i.e., asthma, dyspnoea, or pneumonia), followed by malaise and tremors [17]. The PERS wearable allows the wearer to simply press a button, making the rescue call quicker and safer, along with the presence of trained staff at the centralised point of contact. The rapid dispatch of ambulance or personnel assisting the person at home may have potentially avoided falls [29,30].

Non-fallers performed significantly more calls, including for non-medical events (support calls and service demands), compared to fallers. Even if not significant, non-fallers were less supported by a caregiver. This could have exposed non-fallers to loneliness, explaining the higher number of support calls and service demands. As highlighted by Nymann et al., [31] users who lived alone, experienced more social isolation, and were not supported by a caregiver presented a higher level of self-reported use of the PERS. The possibility for users to reach the TOC for support calls (in addition to the bi-weekly routine calls), may have alleviated the sense of loneliness, a well-known potential risk factor for falls. In fact, the users often carried out these calls due to the desire to talk to someone [17]. In addition, performing support calls showed a fall-risk reduction of 74% (Adjusted OR = 0.26; 95% CI 0.14–0.49) in the non-fallers group. Moreover, the absence of a caregiver could have led non-fallers to use the PERS to address their needs, performing calls for service demands. The support to attend medical examinations, besides guaranteeing safer transportation, ensured the compliance with treatments (e.g., control visits, exams) or a safer response to a problem (e.g., access to the emergency department) preventing a possible worsening of clinical conditions.

On the contrary, recurrent fallers apparently showed evidence of higher frailty: they were significantly less independent, with lower-limb disabilities, however presenting a smaller number of comorbidities. While the risk of falling is influenced by the health status (i.e., the presence of more comorbidities) [27], recurrent falls depend also on a poor or fair self-perceived health conditions [20,32,33,34]. Moreover, they are also associated to a higher request for health services procurement [20]. In our cohort, recurrent fallers performed more support and service demand calls, possibly due to their level of frailty and to the poor self-perceived health, as shown in other studies [20]. However, due to the small sample of recurrent fallers, it was not possible to highlight the presence of any association between recurrent fallers characteristics and the use of PERS.

Our results suggest that wearable technologies, such as PERS, have the potential to support the independent living of older adults, compared to other more complex devices embedded with Artificial Intelligence (AI) or able to monitoring vital signs [35]. Firstly, the possibility for the user to call the TOC makes evident behavioural and physiological patterns of the person’s daily-living that could result in a fall [35]. These could not be visible during the hospitalisation or if the PERS is not connected to a centralised point of contact. This integration between a single centralised point of contact and the device allows a personalised care approach, based on the comprehensive assessment of the user characteristics and needs. The possibility for users to directly interact with the TOC could empower their proactivity in the process of care and fulfilment of their social and health needs [35].

In conclusion, fall-detection technologies have shown a broader use in real world context. As falls result from the combination of intrinsic and extrinsic factors, the combination of new technologies with other types of interventions could be an effective strategy in reducing not only falls, but promoting an independent living [24]. Technology could help healthcare professionals performing a broader evaluation of the fall-risk factors of the person. Most of the available fall-risk instruments do not consider social and economic factors, but they focus only on health status. As in the analysed service, performing two-twice weekly calls allows the TOC staff to be updated to the health, but also to the social and economic issues.

Technology has been introduced in healthcare systems to contribute to fall prevention [36]. Over the last decade, several studies have been focusing on the effectiveness of predictive systems based on gait parameters as a strategy for fall prevention. Even though they are described as promising and useful, they show some inconsistencies to the extent that they cannot be used as a single fall predictive system, regardless of a regular clinical patient evaluation [37,38]. In fact, recent studies on machine learning algorithms have revealed that socio-economical and behavioural factors influence the risk of falling more than health factors [39].

## 5. Implication for Practice

Our results suggest that integrating technological devices with a broader support service tailored to the person may offer a greater advantage in terms of clinical and social outcomes. Future health policies should promote the implementation of different TOCs connected with the social and health services available in a specific district. This could facilitate the integration of services provided to the person (i.e., social services, hospitals, outpatient clinics, etc.), allowing a comprehensive approach to the person’s health.

Promoting policies that support independent living for older adults is fundamental, as ageing is one of the most transformative social phenomena. Future studies should focus on providing tailored interventions designed on individuals’ risk profiles.

## 6. Limits

Our study presents some limitations. Firstly, the sampling of the non-fallers (n = 226) and fallers (≥1 fall, n = 89); single-fallers (n = 66) and recurrent fallers (n = 23) is too unbalanced for meaningful comparisons. Thus, the findings should be considered with caution. Health information (e.g., comorbidities, drugs, using a walking aid, etc.) was collected and self-reported while installing the device. Thus, the number of medications and comorbidities might be underestimated, especially for users who are partially independent and not supported by a caregiver or a home-care service.

We considered only falls detected by the PERS or reported by the users, so they might be underestimated. The small sample could also affect the generalisability of our results, especially for recurrent fallers. In addition, due to the lower number of subjects who fell recurrently, it was impossible to perform a logistic regression model to assess the association between characteristics and real events and recurrent falls.

## 7. Conclusions

Older age is associated with the presence of different health and social conditions that act as risk factors for falls. Consequently, highlighting differences in risk-factors between non-fallers and fallers is challenging. When included in a telemergency service, a PERS could be a valid instrument for meeting the needs of older adults. The benefit of asking for support for any need seems to decrease the risk of falling, possibly interacting with different mechanisms. However, due to the small sample, it was not possible to understand how the use of a PERS could influence the occurrence of recurrent falls in home-care settings.

## Figures and Tables

**Table 1:** Characteristics of non-fallers and fallers.

Variables	Total (n = 315)	Non-Fallers (n = 226; 71.7%)	Fallers (n = 89; 28.3%)	Statistics
Age ^1^				*p* = 0.04
Median (IQR)	86.6 (78.6–91.5)	86 (77.3–91.1)	87.7 (81.8–92.1)	
Sex (Female) n (%)	237 (75.2%)	166 (73.5%)	71 (79.8%)	*p* = 0.24
Marriage status n (%)				*p* = 0.76
Single	62 (19.7%)	47 (20.8%)	15 (16.9%)	
Married	35 (11.1%)	25 (11.1%)	10 (11.2%)	
Divorced	22 (7%)	17 (7.5%)	5 (5.6%)	
Widowed	196 (62.2%)	137 (60.6%)	59 (66.3%)	
Living condition (Alone) n (%)	258 (81.9%)	184 (81.4%)	74 (83.1%)	*p* = 0.72
Caregiver (Yes) n (%)	265 (84.1%)	188 (83.2%)	77 (86.5%)	*p* = 0.47
Walking aid (Yes) n (%)	137 (43.5%)	91 (40.3%)	46 (51.7%)	*p* = 0.07
Independence (Yes) n (%)	126 (40%)	94 (41.6%)	32 (36%)	*p* = 0.36
Weight ^1^				*p* = 0.65
Median (IQR)	68 (58–80)	68 (57.5–80)	68.5 (58–80)	
Comorbidities				*p* = 0.67
Median (IQR)	1 (1–3)	2 (2–3)	3 (2–4)	
Hearing impairment (Yes) n (%)	145 (46%)	101 (44.7%)	44 (49.4%)	*p* = 0.45
Visual impairment (Yes) n (%)	161 (51.1%)	116 (51.3%)	45 (50.6%)	*p* = 0.90
Lower-limb disabilities (Yes) n (%)	118 (37.5%)	84 (37.2%)	34 (38.2%)	*p* = 0.86
Fall-risk increasing drugs (Yes) n (%)	190 (60.3%)	141 (62.4%)	49 (55.1%)	*p* = 0.23
Public users (Yes) n (%)	214 (67.9%)	152 (67.3%)	62 (69.7%)	*p* = 0.68
Number of real events *				*p* < 0.01
Median (IQR)	1 (1–3)	2 (1–3)	1 (0–2)	
Real events n (%)				
Medical problems (Yes) n (%)	93 (29.5%)	75 (33.2%)	18 (20.2%)	*p* = 0.02
Support calls (Yes) n (%)	112 (35.6%)	93 (41.1%)	19 (21.3%)	*p* < 0.01
Service demands (Yes) n (%)	173 (54.9%)	137 (60.6%)	36 (40.4%)	*p* = 0.02

^1^ Age and weight were not available for one participant. Abbreviations: IQR = Interquartile Range. * Excluding falls.

**Table 2:** Logistic regression analysis of real alerts associated with falls.

	Crude Model			Adjusted Model *		
Factors	β (SE)	OR (95% CI)	*p*-Value	β (SE)	OR (95% CI)	*p*-Value
Number of real events	−0.054 (0.156)	0.95 (0.87–1.03)	0.19	-	-	-
Medical problems (Yes)	−0.673 (0.299)	0.51 (0.28–0.91)	0.02	−1.161 (0.344)	0.31 (0.16–0.61)	<0.001
Support calls (Yes)	−0.946 (0.292)	0.38 (0.22–0.69)	0.001	−1.351 (0.329)	0.26 (0.14–0.49)	<0.001
Service demands (Yes)	−0.818 (0.255)	0.44 (0.27–0.73)	0.001	−1.221 (0.301)	0.30 (0.16–0.53)	<0.001

* Adjusted for age, medical problems, support calls and service demands. Abbreviations: SE = Standard Error; OR = Odds Ratio; CI = Confidence Interval.

**Table 3:** Characteristics of fallers and recurrent fallers.

Variables	Total	Single Fallers	Recurrent-Fallers	Statistics
	(n = 89)	(n = 66; 74.1%)	(n = 23; 25.9%)	
Age *				*p* = 0.78
Median (IQR)	87.7 (81.8–92.1)	87.5 (81.8–91.9)	89.1 (80.4–94.7)	
Sex (Female) n (%)	71 (79.8%)	52 (78.8%)	19 (82.6%)	*p* = 0.69
Marriage status n (%)				*p* = 0.58
Single	15 (16.8%)	12 (18.2%)	3 (13%)	
Married	10 (11.2%)	6 (9.1%)	4 (17.4%)	
Divorced	5 (5.6%)	3 (4.5%)	2 (8.7%)	
Widowed	59 (66.3%)	45 (68.2%)	14 (60.9%)	
Living condition (Alone) n (%)	74 (83.1%)	56 (84.8%)	18 (78.3%)	*p* = 0.47
Caregiver (Yes) n (%)	77 (86.5%)	57 (86.4%)	20 (87%)	*p* = 0.94
Walking aid (Yes) n (%)	46 (51.7%)	32 (41.5%)	14 (60.9%)	*p* = 0.31
Independence (Yes) n (%)	32 (36%)	28 (42.4%)	4 (17.4%)	*p* = 0.03
Weight *				*p* = 0.90
Median (IQR)	68.5 (58–80)	69.5 (58–80.1)	67.5 (57.8–80.4)	
Comorbidities				*p* < 0.01
Median (IQR)	3 (2–4)	3 (2–4)	2 (1–2.8)	
Hearing impairment (Yes) n (%)	44 (49.4%)	33 (50%)	11 (47.8%)	*p* = 0.86
Visual impairment (Yes) n (%)	45 (50.6%)	34 (51.5%)	11 (47.8%)	*p* = 0.76
Lower—limb disabilities (Yes) n (%)	34 (38.2%)	21 (31.8%)	13 (56.5%)	*p* = 0.04
Fall—risk increasing drugs (Yes) n (%)	49 (55.1%)	37 (56.1%)	12 (52.2%)	*p* = 0.75
Public users (Yes) n (%)	62 (69.7%)	46 (69.7%)	16 (69.6%)	*p* = 0.99
Number of real events **				*p* = 0.17
Median (IQR)	1 (0–2)	0.5 (0–2)	1 (0–3)	
Real events n (%)				
Medical problems (Yes) n (%)	18 (20.2%)	9 (13.6%)	9 (39.1%)	*p* = 0.01
Support calls (Yes) n (%)	19 (21.3%)	14 (21.2%)	5 (21.7%)	*p* = 0.96
Service demands (Yes) n (%)	36 (40.4%)	25 (37.9%)	11 (47.8%)	*p* = 0.40
Traumatic fall (Yes) n (%)	25 (28.1%)	18 (27.3%)	7 (30.4%)	*p* = 0.77

* Age and weight were not available for one participant. Abbreviations: IQR = Interquartile Range. ** Excluding falls.

## Data Availability

Data that support the finding of our study are available from the Ass.I.S.Te scs company, but restrictions are applied on the availability of these data, which were used thanks to an agreement with the company. The data is not available to the public without the consent of PI Elena Casabona and Ass.I.S.Te.

## Supplementary Materials

The following supporting information can be downloaded at: https://www.mdpi.com/article/10.3390/geriatrics9030069/s1, Table S1: Univariable analysis of the factors associated with falls.
