# Supplementary material for: Factors Associated with Falls in Community-Dwelling Older Adults: A Subgroup Analysis from a Telemergency Service
Source: Geriatrics (Basel). 2024 May 29;9(3):69. doi: 10.3390/geriatrics9030069 (PMC11203033; doi:10.3390/geriatrics9030069)
Supplement: Supplementary file 1 [file geriatrics-09-00069-s001.zip › geriatrics-2972303-supplementary.pdf]

Supplementary Material

Supplementary Table S1. Univariable analysis of the factors associated with falls.

**Factors**	**β (SE)**	**OR**	**95% CI**	***p*-value**
Age	0.028 (0.013)	1.03	1.002-1.056	0.03
Sex (Female)	0.355 (0.304)	1.43	0.79-2.59	0.24
Marriage status				
Married	Ref			
Single	0.225 (0.477)	1.25	0.49-3.19	0.64
Divorced	0.307 (0.632)	1.36	0.39-4.69	0.63
Widowed	-0.074 (0.405)	0.93	0.42-2.06	0.85
Living condition (Alone)	0.119 (0.331)	1.13	0.59-2.15	0.72
Caregiver (Yes)	0.260 (0.360)	1.30	0.64-2.61	0.47
Walking aid (Yes)	0.462 (0.252)	1.59	0.97-2.60	0.07
Independence (Yes)	-0.238 (0.259)	0.79	0.47-1.31	0.36
Weight	0.001 (0.008)	1.00	0.98-1.02	0.86
Comorbidities	0.021 (0.080)	1.02	0.87-1.19	0.80
Hearing impairment (Yes)	0.191 (0.251)	1.21	0.74-1.98	0.45
Visual impairment (Yes)	-0.031 (0.250)	0.97	0.59-1.58	0.90
Lower – limb disabilities (Yes)	0.044 (0.258)	1.05	0.63-1.73	0.86
Fall – risk increasing drugs (Yes)	-0.303 (0.254)	0.74	0.45-1.21	0.23

Abbreviations: SE, Standard Error; OR, Odd Ratio; CI, Confidence Interval.
